# Regulatory effect of microRNA-135a on the Th1/Th2 imbalance in a murine model of allergic rhinitis

**DOI:** 10.3892/etm.2014.1855

**Published:** 2014-07-21

**Authors:** YANYUN LUO, YUQIN DENG, ZEZHANG TAO, SHIMING CHEN, BOKUI XIAO, JIE REN, ZHE CHEN, JIBO HAN, YONGGANG KONG, YU XU, MINJIE DENG

**Affiliations:** Department of Otolaryngology - Head and Neck Surgery, Renmin Hospital of Wuhan University, Wuhan, Hubei 430060, P.R. China

**Keywords:** allergic rhinitis, GATA-3, microRNA, mimic, T helper cell 1/T helper cell 2 imbalance

## Abstract

Allergic rhinitis (AR) is primarily caused by a T helper cell (Th)1/Th2 imbalance. In a murine AR model of a previous study, the serum ovalbumin (OVA)-sIgE concentration was high, whereas microRNA (miR)-135a was lowly expressed in the nasal mucosa. The abnormal expression pattern of miR-135a coincided with highly expressed endogenous factors, including GATA binding protein (GATA)-3 and interleukin (IL)-4, and lowly expressed factors, including T-box expressed in T cells (T-bet) and interferon (IFN)-γ. We hypothesized that miR-135a may play an important role in immune regulation in AR mice. In the present study, AR was induced by OVA in the mice. Two groups of the AR mice were treated with a miR-135a mimic and a mimic control, respectively. The serum and nasal mucosa were collected for analysis. Following miR-135a application, the serum OVA-sIgE concentration was significantly reduced. In the nasal mucosa, the expression levels of miR-135a were higher, the mRNA and protein expression levels of GATA-3 and IL-4 were lower, and the expression levels of T-bet and IFN-γ were higher. The miR-135a corrected the Th1/Th2 imbalance in the AR mice. Findings of this study may provide a basis for novel genetic treatments in addressing allergic diseases.

## Introduction

Allergic rhinitis (AR) is a common disease that affects the quality of life and induces other risk factors, including asthma, termed as *‘*one airway, one disease*’*. The key mechanism which causes AR is the differentiation imbalance of two T-helper cell (Th) subsets, Th1/Th2, which are CD4^+^ helper T lymphocytes, as Th2 presents a higher immune response than Thl (1–5). T-box expressed in T cells (T-bet) and interferon (IFN)-γ are the main Thl-specific transcription factors and cytokines, and GATA binding protein (GATA)-3 and interleukin (IL)-4 are the main Th2-specific transcription factors and cytokines. They play important roles in the differentiation of Th0 cells into Thl and Th2 cells (6–13). As GATA-3 is a Th2-specific transcription factor and plays a pivotal role in the allergic immune response, it is considered as an important therapeutic target for treating allergic diseases (7–8,10–12,14–15).

MicroRNAs (miRNAs or miRs) are a class of small molecule RNAs, which play an important role in the regulation of gene expression at the post-transcriptional level (16). MiRNAs bind to the 3′ UTR (3′ untranslated region) of target genes and inhibit protein translation, by regulating the expression levels of target genes (17). Numerous studies have shown that miRNA regulates proliferation, differentiation and immune function in a variety of types of immune cell within the innate and adaptive immune systems (18–38). The roles of individual types of miRNA in the immune system have also been investigated, including those of miR-21 (21–22), miR-126 (23), miR-133a (24,25), miR-145 (26), miR-146a (27), miR-150 (28), miR-155 (29–37) and miR-181a (38). Among them, only miR-21 (21), miR-126 (23) and miR-155 (34–37) were demonstrated to play a regulatory role in balancing the Thl/Th2 immune response. A deficiency in miR-21 may cause an increase in the levels of IL-12 in the allergic airway by targeting IL-12p35, an increase in the levels of IFN-γ and a reduction in the levels of IL-4 through the IL-12/IFN-γ pathway, thus causing divergence in enhanced cell differentiation, characteristic of a Th1 immunological reaction (21). MiR-126 upregulates the expression levels of the gene POU domain class 2 associating factor 1. This gives rise to high expression levels of the transcription factor PU.1 and low expression levels of GATA-3, thereby inhibiting the secretion of the Th2 cytokines, IL-4, IL-5 and IL-13, in the airway of an asthma mouse model and relieving the airway allergic inflammatory reaction (23). MiR-155 has been demonstrated to act in the regulation of Thl/Th2 differentiation and proliferation. Lack of miR-155 led to the enhancement of its target transcription factor c-Maf, causing favorable differentiation of Th0 cells into Th2 cells, with enhanced production levels of the Th2 cytokine, IL-4 (34). Lack of miR-155 also increased another target, suppressor of cytokine signaling 1 (SOCS1). The increased levels of SOCS1 protein thus inhibited IL-12 and IFN-γ to block Th1 cell differentiation, but promoted Th2 cell differentiation (35–36). Conversely, overexpression of miR-155 caused the differentiation of Th0 cells into Th1 cells. Furthermore, repression of miR-155 expression increased the expression levels of another target, IFN-γ receptor α chain (Rα), suggesting that IFN-γRα inhibited the response of Th1 cells to the antiproliferative effects of IFN-γ (37).

In a previous study, an online bioinformatics program, which predicts and analyzes immune target genes of miRNAs, indicated that a single type of miRNA may be able to control immune regulation through differentiation divergence favoring Th2 cells via GATA-3 target (16). As yet, no studies have confirmed this prediction. Therefore, the present study analyzed the 3′ UTR of mouse GATA-3 and miR-135 with TargetScan. This study may provide a basis for miRNAs to be applied in novel genetic treatments (39) for AR and other allergic diseases.

## Materials and methods

### Experimental animals

Specific pathogen-free male BALB/c mice, aged 5–6 weeks with a weight of 15–18 g, were purchased from the Experimental Animal Center, Renmin Hospital of Wuhan University (Wuhan, China). The animal experiments were approved by the Animal Ethics Committee, Renmin Hospital of Wuhan University. All mice were maintained under standard conventional conditions including mild light (dark), temperature (18–22°C) and humidity (50–60%), with food and water *ad libitum*.

### Preparation of the AR mouse model

The mice were injected intraperitoneally for primary sensitization and then they were treated intranasally following primary sensitization for local stimulation and secondary immunization. The AR group mice were injected intraperitoneally with 400 μl saline containing 10 μg ovalbumin (OVA) and 2 mg aluminum hydroxide (Sigma-Aldrich, St. Louis, MO, USA) on days 1 and 8 to promote drug sensitization. Between days 15 and 21, the mice were administered intranasal drops of 20 μl saline containing 200 μg OVA each day to continuously challenge AR. At the same time, the control group mice were injected intraperitoneally or treated intranasally with an identical dose of saline (400 μl for sensitization on day 1 and 8; 20 μl for continuous challenge each day by the same operator and transferpettor).

### AR intervention by a miR-135a mimic and mimic control

The mimic and mimic control group mice were intraperitoneally sensitized and intranasally challenged with OVA, similar to that of the AR group. On days 14, 16, 18 and 20, the mimic group was treated intranasally with 20 μl saline containing 50 μg miR-135a mimic to intervene with the incidence of AR, while the mimic control group was treated under identical conditions with an arbitrary sequence as previously described (23). The mimic is a cholesterol-conjugated mature miRNA mimic. The miR-135a mimic sequence was as follows: 5′-UAUGGCUUUUUAUUCCUAUGUGA-3′; and the the mimic control sequence was as follows: 5′-UUUGUACUA CACAAAAGUACUG-3′ (Guangzhou RiboBio Co., Ltd., Guangzhou, China).

### Detection of the serum OVA-sIgE concentration by an ELISA

Blood was extracted from the orbital venous plexus of the anesthetized mice 24 h after the last nasal cavity challenge. The blood was left to stand for 20 min prior to 10 min centrifugation at 700 × g. The upper serum was collected and stored at −20°C. The serum concentration of OVA-sIgE was detected by an ELISA (R&D Systems, Minneapolis, MN, USA).

### TargetScanMouse prediction

**Figure 1:**
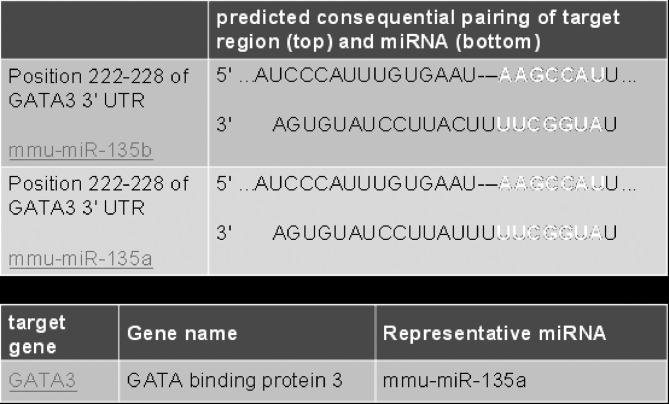
The predicted consequential pairing of target regions and miRNAs. Top: the predicted target region; Bottom: the predicted miRNA.

TargetScanMouse, an online bioinformatics program, was used analyze the 3′ UTR of GATA-3 and the miR-135a 3′ UTR, and thus to predict the consequential pairing of the target region and miRNA (Fig. 1) according to the method described previously (16).

### Quantitative polymerase chain reaction (qPCR)

The anesthetized mice were euthanized by cervical dislocation under sterile conditions. The center seam of the nose from the vertical hard palate of each mouse was excised and sectioned. The nasal septum and mucous membrane of each bilateral nasal cavity lateral wall was eviscerated and placed into sterile vials for storage in liquid nitrogen. The RNA extraction with TRIzol (Invitrogen, Carlsbad, CA, USA) was performed strictly according to the manufacturer’s instructions, and the product was treated with DNase digestion. In the nasal mucosa of the control, mimic, mimic control and AR groups, Bulge-Loop™ miRNA qPCR was used to detect the miR-135a expression levels, and U6 small nuclear RNA was used as an internal control (Guangzhou RiboBio Co., Ltd.). SYBR-Green qPCR was used to detect the relative mRNA expression levels of T-bet, GATA-3, IFN-γ and IL-4. β-actin was used as an internal control (Fermentas, Waltham, MA, USA). The qPCR results used 2^−ΔΔCT^ to represent the relative mRNA expression levels of the target genes. The primer sequences used were: F, 5′-CCTGGACCCAACTGTCAACT-3′ and R, 5′-AACTGT GTTCCCGAGGTGTC-3′ for T-bet; F, 5′-CTGGAG GAGGAACGCTAATG-3′ and R, 5′-AGATGTGGCTCA GGGATGAC-3′ for GATA-3; F, 5′-ACTGGCAAAAGGATG GTGAC-3′ and R, 5′-GACCTGTGGGTTGTTGACCT-3′ for IFN-γ; F, 5′-TCAACCCCCAGCTAGTTGTC-3′ and R, 5′-AAATATGCGAAGCACCTTGG-3′ for IL-4; F, 5′-CAC GATGGAGGGGCCGGACTCATC-3′ and R, 5′-TAAAGA CCTCTATGCCAACAC AGT-3′ for β-actin.

### Western blotting

Western blot analysis was used to detect the relative protein expression levels of T-bet, GATA-3, IFN-γ and IL-4 within the nasal mucosa of the control, mimic, mimic control and AR groups. The relative protein expression levels of the target genes were indicated on the gray scale ratios between each target gene and GAPDH (T-bet antibody sc-21003, GATA-3 antibody sc-9009, IFN-γ antibody sc-59992, and IL-4 antibody sc-1260; Santa Cruz Biotechnology, Inc., Santa Cruz, CA, USA).

### Statistical analysis

Data are presented as the mean ± standard deviation. SPSS statistical software, version 17.0 (SPSS, Inc., Chicago, IL, USA) was used for statistical analysis. Differences between groups were compared using Student’s t-test or analysis of variance. P<0.05 was considered to indicate a statistically significant difference.

## Results

### Low expression levels of miR-135a in the nasal mucosa of AR mice

**Figure 2:**
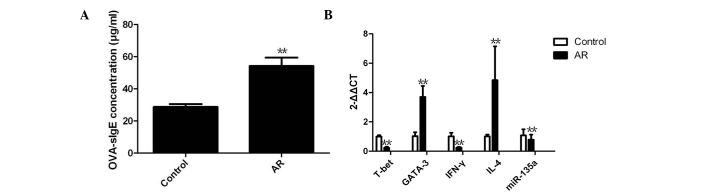
Expression levels of miR-135a in the nasal mucosa of AR mice. AR was induced in the mice by intraperitoneal injection and intranasal drops of OVA. (A) The serum OVA-sIgE concentrations were detected in the AR and control groups by an ELISA 24 h after the last nasal cavity challenge. (B) SYBR Green qPCR was used to assay the mRNA expression levels of T-bet, GATA-3, IFN-γ and IL-4 in the nasal mucosa of the two groups. Bulge-Loop™ miRNA qPCR was used to assay for the relative expression levels of miR-135a of the nasal mucosa in the two groups. (n=10–20 mice/group), ^**^P<0.01. Data were averaged by two independent experiments. OVA, ovalbumin; AR, allergic rhinitis; T-bet, T-box expressed in T cells; GATA, GATA binding protein; IFN, interferon; IL, interleukin; miR, microRNA; qPCR, quantitative polymerase chain reaction.

Following intraperitoneal sensitization and intranasal challenge by OVA, the AR group mice elicited symptoms similar to the clinical occurrence of AR, i.e., nose scratching, sneezing and watery nasal discharge. The control group mice elicited no symptoms. The serum OVA-sIgE concentration in the AR group was significantly elevated compared with that of the control group (Fig. 2). The mRNA expression levels of T-bet and IFN-γ were significantly reduced and those of GATA-3 and IL-4 were significantly increased in the AR mice compared with those in the control group (Fig. 2).

By using Mouse, it was found that the sequence 5′-AAGCCAU-3′ of the 3′ UTR of GATA-3, at the sites of 222–228, had specific binding sites with the 3′-UUCGGUA-5′ of miR-135a. It was also found that the targets of the miR-135a 3′ UTR included the 3′ UTR of GATA-3. The expression levels of miR-135a in the nasal mucosa of the AR mice were significantly lower than those of the control group (Fig. 2).

### Low serum OVA-sIgE concentration in mice following AR intervention using a miR-135a mimic

**Figure 3:**
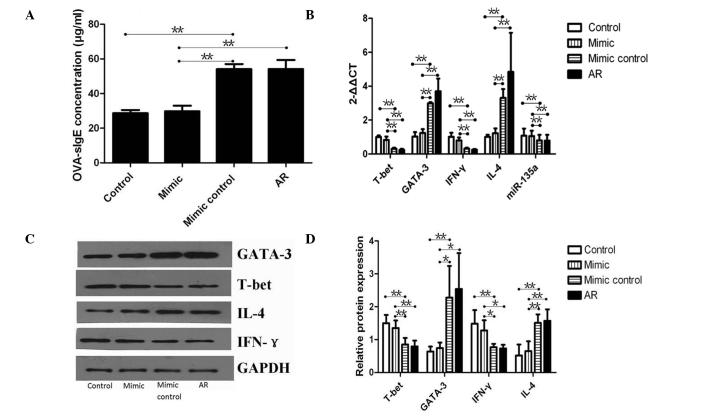
Regulatory effect of a miR-135a mimic on AR. The mimic and mimic control group mice were intraperitoneally sensitized and intranasally challenged by OVA, similar to that of the AR group. (A) The serum OVA-sIgE concentrations were detected by an ELISA in the control, mimic, mimic control and AR groups 24 h after the last nasal cavity challenge. (B) In the nasal mucosa of the four groups, Bulge-Loop™ miRNA qPCR was used to detect the relative miR-135a expression levels; and SYBR-Green qPCR was used to assay for the relative mRNA expression levels of T-bet, GATA-3, IFN-γ and IL-4. (C–D) Western blot analysis was used to detect the relative protein expression levels of T-bet, GATA-3, IFN-γ and IL-4. (n=10–20 mice/group), ^*^P<0.05, ^**^P<0.01. Data were averaged by two independent experiments. OVA, ovalbumin; AR, allergic rhinitis; T-bet, T-box expressed in T cells; GATA, GATA binding protein; IFN, interferon; IL, interleukin; miR, microRNA; qPCR, quantitative polymerase chain reaction.

Following intervention with a miR-135a mimic and a mimic control, it was demonstrated that the serum OVA-sIgE concentration in the mimic group was significantly lower than that in the mimic control and AR groups, but with no significant difference to that of the control group. No significant difference in the serum OVA-sIgE concentrations between the mimic control and AR groups was observed. However, the mimic control and AR groups exhibited significantly higher concentrations of serum OVA-sIgE compared with that of the control group (Fig. 3).

### High mRNA and protein expression levels of miR-135a and low mRNA and protein expression levels of GATA-3 and IL-4 in the nasal mocosa following miR-135a mimic intervention

By using a miR-135a mimic and a mimic control in the nasal mucosa of the mice, the expression levels of miR-135a were significantly higher, and the expression levels of GATA-3 and IL-4 were significantly lower in the mimic group than those of the mimic control and AR groups. However, no significant difference in the levels of them between the mimic and control groups was observed. The expression levels of miR-135a, GATA-3 and IL-4 in the nasal mucosa of the mimic control group were not significantly different from those of the AR group. However, the mimic control and AR groups had significantly lower expression levels of miR-135a and higher expression levels of GATA-3 and IL-4 compared with those of the control group (Fig. 3).

### High mRNA and protein expression levels of T-bet and IFN-γ in the nasal mucosa following miR-135a mimic intervention

It was also demonstrated that the mRNA and protein expression levels of T-bet and IFN-γ in the nasal mucosa of the mimic group were significantly higher than those of the mimic control and AR groups. Between the mimic and control groups, the difference in the levels of T-bet and IFN-γ was not significant. The mRNA and protein expression levels of T-bet and IFN-γ in the nasal mucosa of the mimic control group were not significantly different compared with those of the AR group. The mimic control and AR groups exhibited significantly lower expression levels than those of the control group (Fig. 3).

## Discussion

AR is a disease with symptoms including an itchy nose, sneezing and watery nasal discharge. In the present study, AR mice were induced by intraperitoneal injection and intranasal OVA exposure. Compared with those of the control group, the mice in the AR group elicited symptoms of nose itching, sneezing and watery nasal discharge, along with an increase in the concentration of the serum OVA-sIgE. A previous studied showed that the mRNA expression levels of T-bet and IFN-γ were lower, whereas the mRNA expression levels of GATA-3 and IL-4 were higher in the nasal mucosa of AR mice compared with those of normal mice, thus inhibiting the Th1 immune response and enhancing the Th2 immune response, respectively (40). The mRNA imbalance of T-bet/GATA-3 and IFN-γ/IL-4 results in an imbalance of the Thl/Th2 immune response (4,41), which leads to the occurrence of AR. Enhancing the Th1 and/or reducing the Th2 immune response to correct the Thl/Th2 imbalance may prevent allergic inflammatory reactions and ease the symptoms of allergic diseases (2–3,5,13–15). Regarding its important regulatory role in Th2 cell differentiation and Th2 cytokine secretion, GATA-3 is considered to be an important target for allergic diseases (7–8,10–12,14–15).

To investigate the immune regulatory effects of miRNAs on Thl/Th2 cell differentiation, a previous study used computer bioinformatics to forecast and analyze possible target genes of miRNAs in the immune system and identified that the Th2-specific transcription factor GATA-3 was a potential candidate, owing to its ability to combine with one or several miRNAs (16). The results indicated one or several miRNA candidates were involved in the regulation of Th2 cell differentiation through GATA-3. In the present study, it was demonstrated that GATA-3 and miR-135a were specifically binding to each other, suggesting that miR-135a may play a role in affecting the Th1/Th2 imbalance in AR mice. To test this hypothesis, a miR-135a mimic and a mimic control were used in the present study. It was demonstrated that the miR-135a mimic reduced the serum OVA-sIgE concentration, and downregulated the mRNA and protein levels of GATA-3 and IL-4. Furthermore, the miR-135a mimic caused the mRNA and protein levels of T-bet and IFN-γ to increase in the nasal mucosa. These results further indicated that treatment with miR-135a was effective for correcting the Th1/Th2 imbalance in AR mice.

Overall, this study showed that a miR-135a mimic corrected the imbalance of Th1/Th2 in the nasal mucosa of AR mice. The mimic also reduced the serum OVA-sIgE concentration. Thus, treatment with miR-135a was effective in AR and may provide a basis for miRNA application towards novel genetic treatments for allergic diseases.
